# Quantitative Tomographic Analysis as a Prognostic Tool in Connective Tissue Disease-Associated Interstitial Lung Disease

**DOI:** 10.3390/diagnostics16101413

**Published:** 2026-05-07

**Authors:** Camila Vilas Boas Machado, Pedro Paulo Teixeira da Silva Torres, Danilo Tadao Wada, Francisco Aristofanes Coelho Sarmento Neto, Maria Carolina de Oliveira Rodrigues, Marcel Koenigkam-Santos

**Affiliations:** 1 Department of Medical Imaging, Hematology and Clinical Oncology, Ribeirao Preto Medical School, University of Sao Paulo, Ribeirao Preto 14015-010, SP, Brazil; camilavbmachado@gmail.com; 2 Hospital Israelita Albert Einstein, Goiania 74150-030, GO, Brazil; pedroptstorres@gmail.com; 3 Clinics Hospital, Ribeirao Preto Medical School, University of Sao Paulo, Ribeirao Preto 14049-900, SP, Brazil; dwada@hcrp.usp.br; 4 Diagnostic Imaging Department, Real Hospital Portugues, Av. Gov. Agamenon Magalhaes, 4760, Paissandu, Recife 52010-902, PE, Brazil; sarmentofrancisco@hotmail.com; 5 Department of Medical Clinics, Ribeirao Preto Medical School, University of Sao Paulo, Ribeirao Preto 14049-900, SP, Brazil; mcarolor@usp.br; 6 Department of Medicine, Bauru Medical School, University of Sao Paulo, Bauru 17012-901, SP, Brazil

**Keywords:** connective tissue disease, interstitial lung disease, chest computed tomography

## Abstract

**Background/Objectives:** Interstitial lung disease (ILD) is the main thoracic manifestation of connective tissue diseases (CTDs) and is associated with high morbidity and mortality. High-resolution computed tomography (HRCT) of the chest is considered the gold standard imaging method for detecting and accurately characterizing ILD. This study aims to perform longitudinal visual and quantitative analyses of HRCT scans of patients with ILD secondary to CTD (ILD-CTD) and to correlate these findings with clinical outcomes (functional decline and death) in order to identify tomographic prognostic markers. **Methods:** This retrospective and longitudinal study included 195 patients with CTD who underwent HRCT at baseline and after two follow-ups. Data collected included initial disease extent and subjective impression of progression, as well as quantitative parameters such as lung volume and mean lung density. Forced vital capacity (FVC) and diffusion capacity of carbon monoxide (DLCO) values were also recorded, along with information on patient outcome. **Results:** Quantitative and visual assessments demonstrated moderate agreement in estimating ILD extent. Variations in lung volume, ground-glass index, pulmonary vascular volume-to-lung volume ratio, and disease extent correlated with subjective perception of disease progression. Lung volume reduction and increased disease extent were associated with FVC decrease at follow-up 1, whereas an increased fibrosis index correlated with reduced FVC at follow-up 2. No quantitative parameter was associated with mortality risk. **Conclusions:** The results support the potential benefit of using quantitative CT analysis as a complementary tool to pulmonary function tests and visual analysis in the longitudinal evaluation of ILD-CTD.

## 1. Introduction

Connective tissue diseases (CTDs) comprise a heterogeneous group of autoimmune disorders characterized by systemic inflammation affecting multiple organs, including the lungs. Thoracic involvement most commonly manifests as interstitial lung disease (ILD), particularly in conditions such as systemic sclerosis (SSc), rheumatoid arthritis (RA), and idiopathic inflammatory myopathies. CTD-associated ILD (CTD-ILD) represents a major cause of morbidity and mortality and is a key determinant of long-term prognosis [1,2,3,4].

High-resolution computed tomography (HRCT) is the reference imaging modality for the detection and characterization of ILD in CTDs, enabling detailed assessment of parenchymal abnormalities and disease extent. Beyond its diagnostic role, HRCT is widely used in longitudinal follow-up due to its ability to detect subtle structural changes over time [5].

Although visual assessment remains the standard approach in clinical practice, semi-quantitative scoring systems are limited by substantial intra- and interobserver variability, which reduces reproducibility and challenges the consistent evaluation of disease progression [6,7,8,9,10,11]. In this context, automated quantitative CT analysis has emerged as a promising alternative, providing objective and reproducible metrics based on density and volumetric parameters. Quantitative approaches have been successfully applied in various pulmonary diseases and have shown increasing potential in ILD for assessing disease extent, monitoring progression, and evaluating treatment response [12,13,14,15].

Despite these advances, the prognostic value of quantitative HRCT parameters in CTD-ILD remains incompletely defined, particularly in comparison with visual assessment and pulmonary function tests (PFTs). Understanding the relationship between imaging-derived metrics, functional decline, and clinical outcomes is essential to define their role in risk stratification and patient management.

We hypothesized that quantitative HRCT parameters would provide additional and more objective prognostic information compared with visual assessment and PFTs, particularly in the longitudinal evaluation of disease progression. To address this, we conducted a retrospective longitudinal study including patients with CTD-ILD undergoing serial HRCT and pulmonary function assessment over a follow-up period of up to 60 months.

The aim of this study was to perform longitudinal visual and quantitative analyses of HRCT scans and to correlate these findings with pulmonary function parameters—specifically forced vital capacity (FVC) and diffusing capacity of the lung for carbon monoxide (DLCO)—as well as clinical outcomes in order to identify imaging-based prognostic markers in CTD-ILD.

## 2. Materials and Methods

### 2.1. Patient Selection

This was a single-center, observational, retrospective and longitudinal study including patients under follow-up at a tertiary university hospital, with a confirmed diagnosis of CTD based on established rheumatology society criteria and evidence of pulmonary involvement on HRCT.

Eligible patients were identified from the institutional radiology department database by searching for HRCT reports issued between January 2010 and January 2022 that included a diagnosis of ILD. Electronic medical records were subsequently reviewed to confirm CTD diagnosis and to exclude cases in which ILD was secondary to other causes or of uncertain etiology, based on multidisciplinary clinical and radiological evaluation. Only patients with CTD-ILD were included. Individuals under 18 years of age were excluded.

Given the retrospective single-center design, selection bias cannot be excluded, particularly due to referral patterns and the inclusion of patients undergoing repeated imaging as part of clinical follow-up.

This study was approved by the institutional ethics committee.

### 2.2. High-Resolution Computed Tomography of the Chest

To evaluate pulmonary involvement at baseline and throughout follow-up, up to three HRCT scans were selected for analysis for each patient. The first scan (‘baseline’) corresponded to the first diagnostic-quality HRCT scan performed, while the second (follow-up 1—FU1) and third (follow-up 2—FU2) scans corresponded to those performed closest to 12 months and 60 months after baseline, respectively.

HRCT scans were performed using 16- or 80-detector-row CT scanners (Brillance Big Bore, Philips, Amsterdam, The Netherlands; Aquilion Prime, Canon, Tokyo, Japan), using a similar protocol and the following typical acquisition parameters: volumetric caudocranial acquisition, 1 mm slice thickness, reference mAs of 150, kVp of 120, and tube rotation time < 1.5 s. Patients were positioned in supine with their arms above their head, whenever possible, and images were acquired during inspiratory and expiratory breath-holds. Intravenous iodinated contrast was generally not administered unless required for unrelated clinical hypotheses (e.g., pulmonary embolism). Subsequently, the acquired images were reconstructed in a volumetric manner, with 1 mm thickness, using a standard soft kernel (for use in mediastinal window) and a sharp kernel (for use in lung and bone windows).

All images were transferred to a picture archiving and communication system (PACS) and analyzed on dedicated workstations. Exams considered to be technically inadequate were excluded from the analysis.

### 2.3. Subjective Analysis of Images

The HRCT images were analyzed individually by two radiologists, one with 4 years and the other with 16 years of experience in thoracic imaging. A third radiologist, with 10 years of experience, resolved cases of disagreement by consensus.

Baseline HRCTs were classified according to the 2018 ATS/ERS/JRS/ALAT consensus [16]. In cases of alternative diagnosis, specific patterns were recorded whenever possible [17].

Disease extent at baseline was estimated visually using a modified version of the classification proposed by Goh et al. [7]. Instead of the original five-zone approach, a simplified three-zone classification was adopted, in which limited disease corresponds to fibrotic opacities restricted to the basal segments of the lower lobes; moderate/indeterminate disease includes involvement beyond the basal segments without significant upper lobe extension; and extensive disease is defined by significant involvement of the upper lobes, particularly posterior segments.

**Figure 1:**
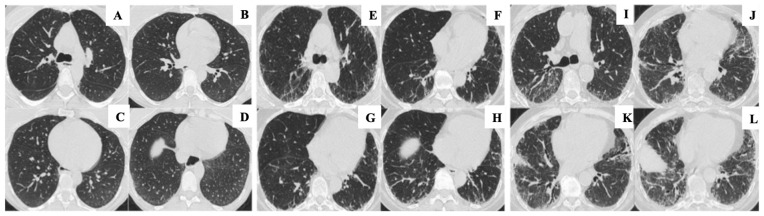
Visual quantification of the extent of pulmonary involvement due to ILD. Images (**A**–**D**) represent limited disease, characterized by involvement predominantly restricted to the basal segments of the lower lobes. Images (**E**–**H**) illustrate moderate/indeterminate disease. Images (**I**–**L**) depict extensive disease, characterized by significant involvement of the upper lobes.

This simplified approach was adopted to improve reproducibility and facilitate longitudinal comparisons in a real-world clinical setting, although it has not been formally validated (Figure 1).

**Figure 2:**
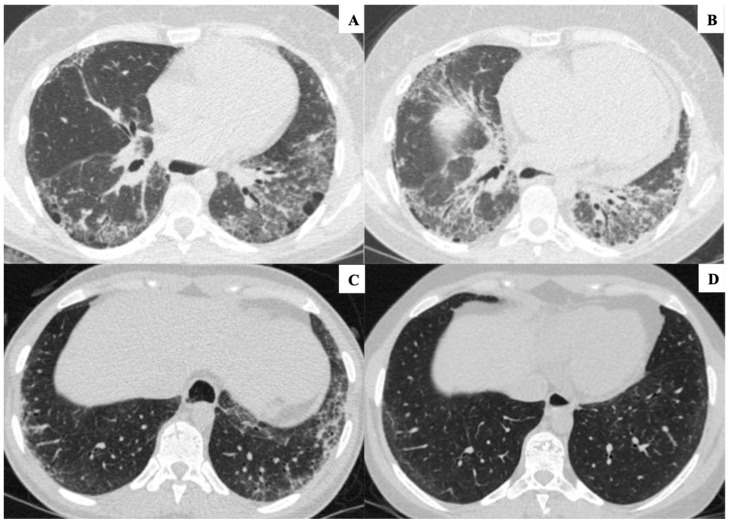
(**A**,**B**): axial HRCT images of a patient at different follow-up times (in (**A**), baseline scan and, in (**B**), FU2 near to 60 months), demonstrating radiological progression of ILD, characterized by increased extent of interstitial opacities and volume loss (in this case, notably in the lower lobes). (**C**,**D**): axial HRCT images of another patient at different follow-up times (in (**C**), baseline scan, and in (**D**), FU2), demonstrating radiological improvement of ILD, characterized by a reduction in interstitial opacities in the lower lung zones, particularly on the left.

For follow-up scans, only changes in disease extent relative to baseline were assessed and categorized as radiological improvement, stability, or progression (Figure 2).

### 2.4. Quantitative Analysis

Quantitative analysis of HRCT images was performed using the scientific software YACTA, version 2.9.0.28. HRCT analysis is based on density-oriented (in Hounsfield Units, HU) image segmentation algorithms combined with various refinement/post-processing mechanisms. In general terms, the program automatically anatomically segments the lungs, pulmonary lobes, airways, and vascular structures and subsequently calculates a series of parameters related to lung parenchymal volume and density, pulmonary vascular volume, and airway thickness [18].

The following quantitative parameters were recorded from the inspiratory images (standard kernel): total lung volume (cm^3^), mean lung density (HU), p90 (the HU value below which 90% of lung voxels are located), fibrosis index (%), ground-glass index (%), R5 (percentage of lung parenchyma with attenuation between −2000 HU and −700 HU), and pulmonary vascular volume. Extent of disease (%), in turn, was estimated by the formula 100%—R5, that is, the percentage of lung parenchyma with attenuation greater than −700 HU, assuming that there is no other superimposed lung pathology (e.g., multiple pulmonary nodules and masses, or infectious processes). Similarly to the visual analysis, ILD was classified as limited when extent of disease was equal to or less than 10%, moderate/indeterminate when extent exceeded 10% but was equal to or less than 30%, and extensive when extent was greater than 30%.

Quantitative CT analysis was performed only on non-contrast-enhanced scans. Density-based quantitative measurements rely on accurate voxel attenuation values, and the presence of intravenous contrast material may artificially increase lung attenuation, potentially affecting the classification of lung voxels based on predefined density thresholds. Therefore, scans acquired after intravenous contrast administration were excluded from the quantitative analysis. In contrast, visual radiologic assessment was performed on all available CT examinations regardless of contrast administration, as the presence of intravenous contrast does not substantially interfere with the visual evaluation of interstitial lung abnormalities or overall disease extent.

### 2.5. Clinical and Pulmonary Function Test Data

Clinical and functional data (FVC and DLCO) were obtained from the patients’ electronic medical records. Sex, age at the start of follow-up, and type of CTD were recorded, as well as data regarding potential loss to follow-up and/or death.

FVC and DLCO values were recorded as percentages of the predicted values. In a manner analogous to the selection of HRCT scans, up to three PFTs were selected for each patient, using the date of the HRCT scan as a reference. To be included in the analysis, the PFTs had to be performed within a maximum interval of six months from the reference HRCT.

FVC variations in the second and third spirometry tests were recorded as a decline or increase equal to or greater than 10%, or variations less than 10%, always using the first test as the reference. Similarly, DLCO variations were recorded as a decline or increase equal to or greater than 15%, or variations less than 15%, also using the initial test as the reference.

### 2.6. Statistical Analysis

Demographic characteristics and qualitative variables were expressed as absolute values and/or percentages, while quantitative variables were presented as mean ± standard deviation.

Longitudinal changes in quantitative CT parameters were analyzed using linear mixed-effects models with random intercepts to account for repeated measurements within individuals across follow-up timepoints. Continuous variables were compared using analysis of variance (ANOVA), followed by Tukey post hoc tests when appropriate, and categorical variables were compared using chi-square or Fisher’s exact tests.

For analyses evaluating associations between quantitative imaging parameters and clinical outcomes, linear regression models were applied. The distribution of quantitative variables was assessed for normality, and variables with skewed distributions were log-transformed to improve model stability. When necessary, a constant was added prior to transformation (e.g., log(Y + 67)) to ensure positive values.

Missing longitudinal data were handled under the assumption of missing at random. Mixed-effects models were chosen as they are robust to unbalanced repeated measures and allow inclusion of patients with incomplete follow-up data.

Agreement between subjective and quantitative assessments, as well as intra- and interobserver agreement, was evaluated using the kappa coefficient.

Descriptive analyses were performed to characterize the actual time intervals between HRCT examinations, summarized using median and interquartile range (IQR).

To assess potential attrition bias, baseline characteristics were compared between patients with complete and incomplete follow-up. Additionally, a sensitivity subgroup analysis comparing systemic sclerosis (SSc) and non-SSc CTDs was conducted to evaluate the consistency of imaging-functional associations across disease subtypes.

All statistical analyses were performed using R software version 4.1.3 and SAS software version 9.4. A *p*-value < 0.05 was considered statistically significant.

## 3. Results

**Figure 3:**
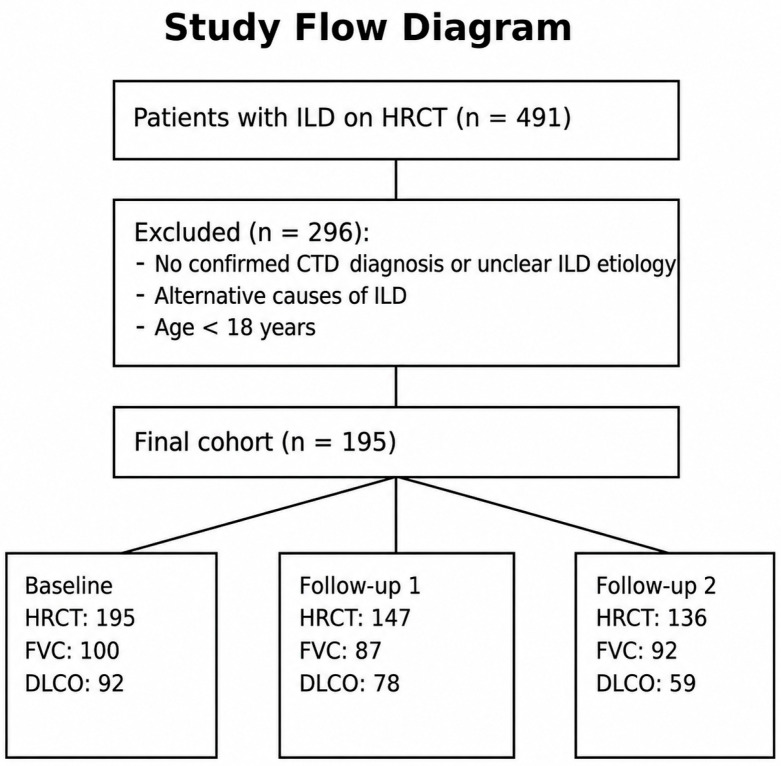
Flow diagram illustrating patient selection and data availability across timepoints. A total of 491 patients with interstitial lung disease (ILD) identified on high-resolution computed tomography (HRCT) were screened. After exclusion of patients without confirmed connective tissue disease (CTD), with alternative causes of ILD, or younger than 18 years, 195 patients were included in the final cohort. The number of patients with available HRCT, forced vital capacity (FVC), and diffusing capacity of the lung for carbon monoxide (DLCO) data at baseline, follow-up 1, and follow-up 2 is shown.

The initial database search identified 491 patients with ILD detected on HRCT. Among these, 251 patients were excluded either because the etiology of ILD had not been investigated (mostly individuals under oncologic follow-up) or because there was no diagnosis of CTD upon medical record review. An additional 41 patients were excluded due to uncertainty regarding whether the pulmonary disease was secondary to the rheumatologic condition or attributable to other factors, such as environmental exposure, drug toxicity, or bronchoaspiration, based on multidisciplinary clinical and radiological evaluation. Lastly, four patients were excluded for being under 18 years of age at the start of follow-up. Thus, the final study sample comprised 195 patients (Figure 3).

The patients included had a minimum age of 19 years and a maximum of 74 years at the beginning of the follow-up, with a mean age of 47.3 years (±12.5 years). Most of the sample (83%) was composed of women, with a female-to-male ratio of 4.7:1.

SSc was the most prevalent CTD in the study group, observed alone in 54% of patients (*n* = 106) or in association with other CTDs such as SLE (*n* = 11), RA (*n* = 4), SS (*n* = 5), or dermatomyositis (*n* = 2). There was also an association between SSc and two other CTDs in two patients, one with SS and dermatomyositis and the other with SS and SLE.

RA was the second most common CTD, diagnosed alone in 32 patients. One patient had juvenile idiopathic arthritis in association with SS. Following in prevalence were idiopathic inflammatory myopathies in 12 patients, SLE and MCTD each in 6 patients, and SS in 5 patients. Finally, three patients were considered to have some CTD, but the specific diagnosis could not be established.

Among the 195 patients included in the study, 18 (9%) were lost to FU1 and 27 (14%) between follow-ups 1 and 2. Fourteen patients (7%) had not yet completed 60 months of follow-up by the end of data collection. The longest follow-up period observed was 108 months. The availability of longitudinal data varied across timepoints, particularly for pulmonary function tests, resulting in an unbalanced dataset for longitudinal analyses.

A total of 25 patients (14%) died, with 4 deaths occurring within 12 months and 21 within 60 months. Among those who died, 8 deaths were attributed to infectious processes, 4 to treatment-related complications, 1 to malignancy, and 1 to malnutrition. The cause of death is unknown for 11 patients.

The actual time intervals between HRCT examinations were also analyzed. The median time between baseline and FU1 was 13.8 months (IQR: 11.8–16.5), while the median time between baseline and FU2 was 56.7 months (IQR: 44.2–61.6).

To evaluate potential attrition bias, baseline characteristics were compared between patients with complete HRCT follow-up (*n* = 115) and those with incomplete follow-up (*n* = 80). The groups were similar regarding sex distribution (84.3% vs. 80.0%, *p* = 0.448), CTD subtype (SSc: 71.3% vs. 68.8%, *p* = 0.751), and baseline FVC% (72.9 ± 17.9 vs. 72.6 ± 17.0, *p* = 0.953). Patients with incomplete follow-up were slightly older (49.6 ± 12.6 vs. 45.7 ± 13.0 years, *p* = 0.036).

### 3.1. Subjective Analysis of HRCT Scans

A total of 478 HRCT scans from this cohort were evaluated. Of the patients included, 59% had three consecutive HRCTs available for evaluation, 27% had two valid exams (baseline and one follow-up), and 14% had only a single scan.

**Figure 4:**
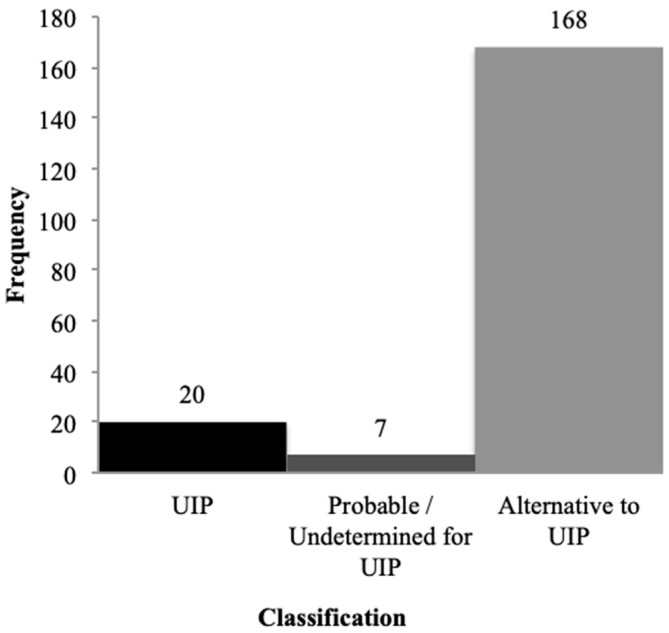
Distribution of HRCTs according to the tomographic patterns proposed in the ATS/ERS/JRS/ALAT consensus (UIP, probable/undetermined for UIP or alternative diagnosis to UIP).

**Figure 5:**
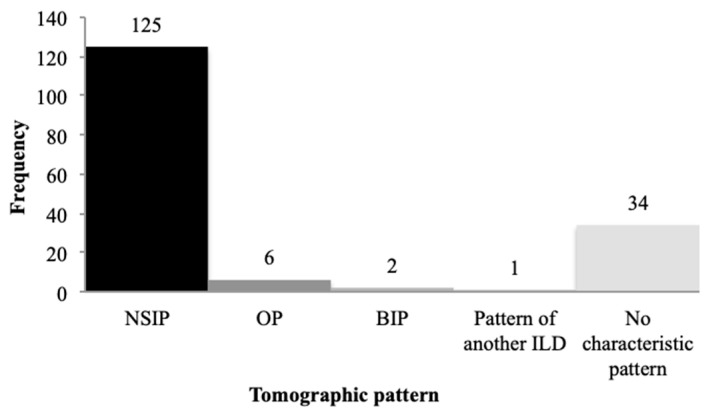
Distribution of tomographic patterns of HRCTs classified as an alternative diagnosis to UIP.

Considering the tomographic patterns proposed in the 2018 ATS/ERS/JRS/ALAT consensus, 86% of patients exhibited an alternative pattern to usual interstitial pneumonia (UIP), 10% met criteria for UIP, and 4% were classified as probable or indeterminate for UIP at baseline scans (Figure 4). Among those with an alternative diagnosis, when specific patterns could be identified, 125 exams (64%) were consistent with nonspecific interstitial pneumonia (NSIP), 6 with organizing pneumonia (OP), and 2 with bronchiolocentric interstitial pneumonia (BIP). One scan demonstrated a pattern of another ILD, and 34 baseline HRCTs were considered unclassifiable (Figure 5).

Most patients (55%) were classified as having moderate/indeterminate disease extent at baseline. Limited disease was found in 25% of the cases, and extensive disease in 20%. At the FU1 HRCT, radiological stability was observed in 50% of patients, improvement in 9%, and progression in 17%. At the FU2 scan, disease stability was observed in 23% of cases, improvement in 9%, and progression in 37%. The relative risk of radiological progression over 5 years was 2.39 times higher than at 1 year.

Inter-observer agreement was considered reasonable, with a kappa coefficient of 0.40 at baseline, 0.26 at FU1, and 0.23 at FU2, indicating decreasing agreement over time.

### 3.2. Quantitative Analysis of HRCT Scans

Among the 478 CT scans selected for visual analysis, 21 were excluded from the quantitative analysis due to intravenous contrast administration, which interferes with parenchymal attenuation measurements. Two additional scans were excluded due to significant segmentation errors during automated processing. Therefore, 455 HRCTs were included in the automated quantification analysis, comprising 181 baseline scans, 141 FU1 scans, and 133 FU2 scans.

Quantitative analysis of initial disease extent revealed moderate/indeterminate disease in 63% of cases and extensive disease in 37%. None of the scans were classified as showing limited disease based on the software’s evaluation.

Longitudinal analysis of ILD-related quantitative parameters was performed using linear mixed-effects models. A significant reduction in lung volume was observed between follow-up timepoints (*p* = 0.02), although no significant differences were found between baseline and FU1 or baseline and FU2. An increase in the fibrosis index was observed at FU2 compared to both baseline and FU1, while a decrease in the ground-glass index was noted between baseline and FU1 (*p* = 0.01). No other parameters demonstrated significant temporal variation.

### 3.3. Pulmonary Function Tests Analysis

Pulmonary function data were obtained from 286 exams; however, data availability was limited, particularly at later follow-up timepoints. Only 19% of patients had three consecutive exams meeting inclusion criteria, and 17% had no eligible exams. This variability in data availability may have influenced the robustness of longitudinal functional analyses.

The mean predicted FVC (±standard deviation) at baseline and follow-ups 1 and 2 was 72.75% (±17.37), 76.07% (±20.14), and 72.84% (±20.75), respectively. In turn, the mean predicted DLCO was 62.04% (±22.42) at baseline, 61.38% (±22.74) at FU1, and 63.59% (±21.50) at FU2.

Among the 195 patients included, 66 had both a baseline and a 1-year follow-up spirometry. Of these, 20% showed a decline in FVC ≥ 10%, 62% had variations in FVC below 10%, and 18% showed an increase in FVC ≥ 10%. The evaluation at 5 years, in comparison with the baseline, was obtained from examinations of 55 patients. FVC declined by ≥10% in 29% of them, while 55% showed changes below 10%, and 16% had an increase of ≥10%.

DLCO progression was analyzed in 56 patients at FU1 and in 27 patients at FU2. After one year, 34% of patients showed a DLCO decline of ≥15%, 50% had changes below 15%, and 16% had an increase of ≥15%. After 5 years, 37% experienced a DLCO decline ≥15%, 33% had changes below 15%, and 30% showed a DLCO increase ≥15% compared to the baseline.

The mean time for FVC decline was 26.70 months (±18.04), while for DLCO decline it was 23.92 months (±16.89).

### 3.4. Subjective Analysis vs. Quantitative Analysis

The concordance between visual and quantitative classification of disease extent at baseline was moderate, with a weighted kappa coefficient of 0.34 (95% CI: 0.25–0.43), indicating limited agreement between methods.

Changes in lung volume, mean lung density, P90, fibrosis index, ground-glass index, pulmonary vascular volume and disease extent, obtained through the software analysis and at follow-ups 1 and 2, relative to the baseline, were compared across groups of patients classified visually as having radiological improvement, stability, or progression of ILD in the same time intervals.

Between baseline and FU1, significant differences were observed in lung volume, pulmonary vascular volume-to-lung volume ratio, and disease extent among patients with radiological improvement, stability, and progression. Significant differences were also found in ground-glass index and pulmonary vascular volume between the improvement and stability groups and between improvement and progression groups. Additionally, a significant difference in mean lung density was noted between the improvement and progression groups. No significant differences were found for P90 or fibrosis index among the three visual evolution groups in this comparison. The results are detailed in Table 1.

In the comparison between baseline and FU2, significant differences were observed in lung volume, P90, and disease extent between patients with radiological improvement and progression and between stability and progression groups. There were also differences in the pulmonary vascular volume-to-lung volume ratio between improvement and stability groups and between improvement and progression groups. Significant differences were noted in mean lung density between improvement and progression groups and in the fibrosis index between stability and progression groups. Lastly, the ground-glass index varied significantly among all three visual categories. These results are summarized in Table 2.

### 3.5. Subjective Analysis vs. Pulmonary Function Tests

The comparison between the increase ≥ 10%, stability (variation between ± 10%) or decline ≥ 10% of FVC and the improvement, stability or progression of ILD in the visual HRCT assessment revealed a statistically significant difference in at FU1 (*p* < 0.01), but not at FU2 (*p* = 0.12).

The comparison between the increase ≥ 15%, stability (variation between ± 15%) or decline ≥ 15% of DLCO and the improvement, stability or progression of ILD in the visual assessment of HRCT did not show any statistically significant difference at any time of follow-up (*p* = 0.22 at 12 months and *p* = 0.69 at 60 months).

### 3.6. Quantitative Analysis vs. Pulmonary Function Test

The variations in lung volume, mean lung density, P90, fibrosis index, ground-glass index, pulmonary vascular volume and disease extent, obtained through quantitative analysis at both follow ups in relation to the baseline, were compared between individuals who presented decline, stability or increase of FVC in the same time intervals.

In the comparison between baseline and FU1, significant differences were observed in changes in mean lung density, P90, and disease extent between patients with decline and stability of FVC and decline and increase in FVC. Differences were also found in changes in lung volume and ground-glass index between patients with decline and increase in FVC. The results are detailed in Table 3.

In the comparison between baseline and FU2, differences were observed in changes in mean lung density, P90, fibrosis index, ground-glass index, pulmonary vascular volume-to-lung volume ratio, and disease extent between patients with decline and increase in FVC. A significant difference was also found in lung volume changes between patients with decline and increase in FVC and stability and increase of FVC. The results are detailed in Table 4.

Using an FVC decline ≥ 10% as the outcome, reduction in lung volume and increase in disease extent were associated with functional decline at FU1 (relative risk of 1.05 and 1.03, respectively; *p* < 0.01). At FU2, an increase in the fibrosis index was associated with FVC decline (relative risk of 1.02; *p* < 0.01).

The variations in lung volume, mean lung density, P90, fibrosis index, ground-glass index, pulmonary vascular volume and disease extent, obtained through quantitative analysis at both follow ups in relation to the baseline, were compared between individuals who presented decline, stability or increase in DLCO in the same time intervals, with no significant difference being observed between the multiple comparisons.

A sensitivity subgroup analysis comparing patients with systemic sclerosis (SSc) and non-SSc CTDs was performed.

At FU1, the associations between reduction in lung volume and increase in disease extent with FVC decline ≥ 10% were preserved in both subgroups. In the SSc group, lung volume reduction and disease extent increase were significantly associated with FVC decline (*p* = 0.002 and *p* = 0.001, respectively), while in the non-SSc group these associations also remained significant (*p* = 0.003 and *p* = 0.022, respectively).

At FU2, the association between fibrosis index increase and FVC decline was not statistically significant in either subgroup, likely reflecting reduced statistical power due to smaller sample sizes.

These findings support the consistency of the main imaging-functional associations despite underlying CTD heterogeneity.

### 3.7. Quantitative Analysis and Pulmonary Function Tests vs. Mortality

Among the four patients who died until FU1, none underwent spirometry or DLCO testing, and only one underwent follow-up HRCT. Therefore, it was not possible to evaluate the behavior of these parameters or their possible relationship with mortality.

Quantitative HRCT analysis, as well as FVC and DLCO assessment, in the remaining 21 patients who died until FU2 demonstrated a significant association only between DLCO decline and mortality (*p* = 0.02), with a relative risk of 1.444 for each 15% decline in DLCO. No significant association was found between any of the other parameters and mortality risk.

Additionally, subgroup analyses according to CTD subtype were performed at baseline and across follow-up timepoints. At baseline, no significant differences were observed in quantitative CT or functional parameters between groups, except for age.

Longitudinal analyses at FU1 and FU2 demonstrated similar patterns of change across all subgroups, including comparable reductions in lung volume and increases in disease extent and fibrosis-related parameters. No statistically significant differences were observed between CTD subtypes at any timepoint.

These findings support the consistency of imaging-functional associations across different CTD subtypes (Supplementary Table S1).

## 4. Discussion

The present study provides a retrospective longitudinal evaluation of pulmonary involvement in connective tissue disease-associated interstitial lung disease (CTD-ILD) using both visual assessment and automated quantitative analysis of high-resolution computed tomography (HRCT). These imaging approaches were compared and correlated with pulmonary function test (PFT) parameters, namely forced vital capacity (FVC) and diffusing capacity of the lung for carbon monoxide (DLCO), to investigate their potential prognostic value.

A central finding of this study was the discrepancy between visual and quantitative classifications of disease extent at baseline. While approximately one quarter of patients were visually classified as having limited disease, none met this criterion when assessed by automated quantitative analysis. This discordance highlights an inherent limitation of density-based quantitative methods when used in isolation and emphasizes the importance of integrating quantitative metrics with visual interpretation and clinical context [19,20,21,22,23]. Importantly, density-based measurements may also be influenced by factors such as lung inflation level, vascular congestion, atelectasis, or concomitant inflammatory or infectious processes [24,25].

Visual HRCT assessment demonstrated that the relative risk of radiological progression over five years was more than twice that observed at one-year follow-up. In parallel, quantitative analysis revealed a significant increase in the fibrosis index at long-term follow-up (FU2), supporting the concept of progressive fibrotic remodeling over time [26,27,28]. In addition, a reduction in the ground-glass index between baseline and FU1 was observed, which may reflect a transition from potentially reversible inflammatory changes to more established fibrotic alterations, a pattern previously associated with worse outcomes in ILD [29,30].

Interobserver agreement for visual assessment was reasonable at baseline but progressively declined at later follow-up examinations, consistent with previous studies demonstrating variability in visual scoring systems [10,31]. This reduction may be explained by the increasing heterogeneity of imaging findings over time, resulting from disease progression, treatment effects, and the coexistence of fibrotic and inflammatory components.

Direct comparison between visual and quantitative assessments demonstrated a systematic tendency for radiologists to underestimate disease extent relative to automated densitometric analysis. The modest agreement observed between the two approaches reinforces the limitations of visual estimation alone, which is inherently dependent on observer experience [22,23]. In contrast, density-based algorithms may capture subtle increases in lung attenuation that are not readily appreciated during visual interpretation, although they may also be influenced by non-parenchymal factors [24,32].

Several quantitative parameters—including lung volume, ground-glass index, pulmonary vascular volume-to-lung volume ratio, and disease extent—showed significant associations with visually assessed radiological improvement, stability, or progression. Conversely, P90, fibrosis index, and absolute pulmonary vascular volume demonstrated fewer consistent differences among visual evolution groups, suggesting that not all quantitative metrics contribute equally to longitudinal disease characterization [33,34].

When correlated with functional outcomes, reductions in lung volume and increases in disease extent were significantly associated with an FVC decline of at least 10% at one-year follow-up. At longer-term follow-up, an increase in the fibrosis index was associated with functional deterioration. Given the established prognostic role of FVC decline in ILD, these findings support the potential utility of selected quantitative HRCT parameters as markers of disease progression [35,36]. Notably, these associations were consistent across CTD subtypes in subgroup analyses, supporting the potential generalizability of the main findings despite the heterogeneity of the underlying diseases.

In contrast, no quantitative imaging parameter demonstrated a robust association with mortality in this cohort. This finding may be partially explained by the limited number of deaths and by the heterogeneous causes of mortality, many of which were not directly related to ILD. DLCO decline, however, emerged as a significant predictor of mortality, in agreement with previous studies highlighting its prognostic relevance in fibrosing lung diseases [33,35].

Advances in quantitative imaging and artificial intelligence have further expanded the potential applications of CT-based analysis beyond ILD. Automated tools have been increasingly explored in lung cancer screening settings and in the detection of interstitial abnormalities in specific populations, including non-smoking individuals, suggesting a broader role for imaging biomarkers in pulmonary disease stratification and early detection [37,38].

This study has several limitations. Its single-center, retrospective design may limit generalizability and introduce selection bias, precluding causal inference. The absence of structured treatment data precluded adjustment for therapeutic effects, which may have influenced both imaging and functional outcomes. In addition, the limited availability of pulmonary function data, particularly at later follow-up timepoints, may have introduced bias in longitudinal analyses. Grouping different CTDs into a single cohort, although necessary to achieve an adequate sample size, may have obscured disease-specific patterns. External validation in independent and multicenter cohorts is warranted to confirm the reproducibility and generalizability of these findings. Finally, the absence of correction for multiple comparisons warrants cautious interpretation of findings with marginal statistical significance.

Overall, these results reinforce the concept that quantitative and visual HRCT analyses should be regarded as complementary rather than interchangeable tools in the longitudinal assessment of CTD-ILD.

## 5. Conclusions

Quantitative analysis of high-resolution computed tomography represents a promising approach for the objective and reproducible assessment of interstitial lung disease associated with connective tissue disorders. In this study, software-derived imaging parameters—particularly lung volume, disease extent, mean lung density, P90, and pulmonary vascular metrics—demonstrated meaningful associations with visual radiological assessment and longitudinal changes in pulmonary function.

Reductions in lung volume and increases in disease extent were associated with early functional decline, while progression of fibrotic metrics correlated with longer-term deterioration. These findings support the integration of quantitative HRCT parameters with visual interpretation and pulmonary function testing to enhance disease monitoring and prognostic stratification in CTD-ILD.

Nevertheless, quantitative CT metrics should not be interpreted in isolation. Methodological variability, differences in acquisition protocols, and the influence of non-parenchymal structures on density-based measurements necessitate cautious interpretation. Prospective, multicenter studies with standardized imaging protocols and comprehensive treatment data are required to validate these findings and to define the role of quantitative HRCT in routine clinical practice.

## Figures and Tables

**Table 1:** Comparison of quantitative analysis parameters among the groups classified as radiological ‘improvement,’ ‘stability,’ or ‘progression’ in the visual assessment of HRCT scans at FU1. 95% CI—95% confidence interval; * log transformation (Y + 67).

Comparisons	Estimated Mean Difference	95% CI	*p*-Value
**Lung volume**			
Improvement vs. Stability	15.29	5.33; 25.24	**<0.01**
Improvement vs. Progression	27.06	15.58; 38.54	**<0.01**
Stability vs. Progression	11.77	3.82; 19.72	**<0.01**
**Mean lung density**			
Improvement vs. Stability	6.16	0.86; 11.46	0.02
Improvement vs. Progression	7.56	1.45; 13.68	**0.01**
Stability vs. Progression	1.40	−2.83; 5.63	0.71
**P90**			
Improvement vs. Stability	5.86	−8.7; 20.43	0.61
Improvement vs. Progression	13.28	−3.51; 30.08	0.15
Stability vs. Progression	7.42	−4.21; 19.05	0.29
**Fibrosis index**			
Improvement vs. Stability	5.54	−10.83; 21.91	0.70
Improvement vs. Progression	−6.23	−25.11; 12.64	0.71
Stability vs. Progression	−11.78	−24.84; 1.29	0.09
**Ground-glass index**			
Improvement vs. Stability	−42.13	−70.39; −13.88	**<0.01**
Improvement vs. Progression	−64.13	−96.71; −31.54	**<0.01**
Stability vs. Progression	−21.99	−44.55; 0.57	0.06
**Pulmonary vascular volume**			
Improvement vs. Stability	−18.74	−33.76; −3.72	**0.01**
Improvement vs. Progression	−21.12	−38.44; −3.8	**0.01**
Stability vs. Progression	−2.38	−14.38; 9.61	0.88
**Pulmonary vascular volume/Lung volume**			
Improvement vs. Stability	−28.41	−42.87; −13.95	**<0.01**
Improvement vs. Progression	−43.10	−59.77; −26.43	**<0.01**
Stability vs. Progression	−14.69	−26.24; −3.15	**<0.01**
Disease extent *			
Improvement vs. Stability	−0.54	−0.76; −0.31	**<0.01**
Improvement vs. Progression	−0.80	−1.06; −0.54	**<0.01**
Stability vs. Progression	−0.26	−0.44; −0.08	**<0.01**

**Table 2:** Comparison of quantitative analysis parameters among the groups classified as radiological ‘improvement,’ ‘stability,’ or ‘progression’ in the visual assessment of HRCT scans at FU2. 95% CI—95% confidence interval.

Comparisons	Estimated Mean Difference	95% CI	*p*-Value
**Lung volume**			
Improvement vs. Stability	9.70	−2.29; 21.68	0.14
Improvement vs. Progression	24.79	13.54; 36.04	**<0.01**
Stability vs. Progression	15.09	6.69; 23.5	**<0.01**
**Mean lung density**			
Improvement vs. Stability	3.56	−2.59; 9.7	0.36
Improvement vs. Progression	7.20	1.44; 12.97	**0.01**
Stability vs. Progression	3.65	−0.66; 7.96	0.11
**P90**			
Improvement vs. Stability	3.35	−14.97; 21.67	0.90
Improvement vs. Progression	19.20	2.01; 36.4	**0.02**
Stability vs. Progression	15.86	3.01; 28.7	**0.01**
**Fibrosis index**			
Improvement vs. Stability	1.40	−17.18; 19.98	0.98
Improvement vs. Progression	−14.85	−32.3; 2.59	0.11
Stability vs. Progression	−16.26	−29.29; −3.23	**0.01**
**Ground-glass index**			
Improvement vs. Stability	−29.36	−56.32; −2.39	**0.03**
Improvement vs. Progression	−63.28	−88.59; −37.96	**<0.01**
Stability vs. Progression	−33.92	−52.83; −15.01	**<0.01**
**Pulmonary vascular volume**			
Improvement vs. Stability	−15.75	−39.35; 7.86	0.26
Improvement vs. Progression	−13.37	−35.54; 8.79	0.33
Stability vs. Progression	2.37	−14.18; 18.93	0.94
**Pulmonary vascular volume/Lung volume**			
Improvement vs. Stability	−22.20	−43.87; −0.54	**0.04**
Improvement vs. Progression	−34.30	−54.65; −13.96	**<0.01**
Stability vs. Progression	−12.10	−27.3; 3.09	0.15
**Disease extent**			
Improvement vs. Stability	−17.23	−35.65; 1.18	0.07
Improvement vs. Progression	−43.46	−60.75; −26.17	**<0.01**
Stability vs. Progression	−26.23	−39.14; −13.31	**<0.01**

**Table 3:** Comparison between the parameters of the quantitative analysis and the groups with ‘decline’ (decline ≥ 10%), ‘stability’ (variation less than 10% above or below) or ‘increase’ (increase ≥ 10%) of FVC at the FU1; 95% CI—95% confidence interval.

Comparisons	Estimated Mean Difference	95% CI	*p*-Value
**Lung volume**			
Decline vs. Stability	−12.37	−26.53; 1.79	0.10
Decline vs. Increase	−26.35	−44.63; −8.07	**<0.01**
Stability vs. Increase	−13.97	−29.27; 1.32	0.08
**Mean lung density**			
Decline vs. Stability	−6.10	−10.07; −2.14	**<0.01**
Decline vs. Increase	−9.10	−14.22; −3.98	**<0.01**
Stability vs. Increase	−3.00	−7.28; 1.29	0.22
**P90**			
Decline vs. Stability	−19.04	−36.55; −1.54	**0.03**
Decline vs. Increase	−23.84	−46.44; −1.25	**0.04**
Stability vs. Increase	−4.80	−23.7; 14.1	0.81
**Fibrosis index**			
Decline vs. Stability	9.16	−16.4; 34.72	0.66
Decline vs. Increase	14.55	−18.44; 47.55	0.54
Stability vs. Increase	5.39	−22.22; 33	0.88
**Ground-glass index**			
Decline vs. Stability	26.53	−0.1; 53.16	0.05
Decline vs. Increase	47.84	13.46; 82.22	**<0.01**
Stability vs. Increase	21.31	−7.45; 50.07	0.18
**Pulmonary vascular volume**			
Decline vs. Stability	−1.49	−21.82; 18.84	0.98
Decline vs. Increase	−2.74	−28.99; 23.51	0.97
Stability vs. Increase	−1.25	−23.21; 20.71	0.99
**Pulmonary vascular volume/Lung volume**			
Decline vs. Stability	11.56	−11.58; 34.71	0.45
Decline vs. Increase	20.96	−8.92; 50.84	0.22
Stability vs. Increase	9.40	−15.6; 34.4	0.64
**Disease extent**			
Decline vs. Stability	25.07	7.86; 42.29	**<0.01**
Decline vs. Increase	36.63	14.4; 58.85	**<0.01**
Stability vs. Increase	11.55	−7.04; 30.15	0.30

**Table 4:** Comparison between the parameters of the quantitative analysis and the groups with ‘decline’ (decline ≥ 10%), ‘stability’ (variation less than 10% above or below) or ‘increase’ (increase ≥ 10%) of FVC at the FU2; 95% CI—95% confidence interval.

Comparisons	Estimated Mean Difference	95% CI	*p*-Value
**Lung volume**			
Decline vs. Stability	−5.71	−16.95; 5.54	0.44
Decline vs. Increase	−20.51	−35.23; −5.78	**<0.01**
Stability vs. Increase	−14.80	−28.24; −1.36	**0.03**
**Mean lung density**			
Decline vs. Stability	−3.32	−7.11; 0.47	0.10
Decline vs. Increase	−6.63	−11.56; −1.71	**<0.01**
Stability vs. Increase	−3.32	−7.83; 1.2	0.19
**P90**			
Decline vs. Stability	−18.32	−36.56; −0.08	0.05
Decline vs. Increase	−35.97	−59.85; −12.08	**<0.01**
Stability vs. Increase	−17.65	−39.46; 4.15	0.13
**Fibrosis index**			
Decline vs. Stability	13.13	−1.71; 27.98	0.09
Decline vs. Increase	23.47	4.04; 42.91	**0.01**
Stability vs. Increase	10.34	−7.4; 28.08	0.34
**Ground-glass index**			
Decline vs. Stability	22.84	−2.85; 48.53	0.09
Decline vs. Increase	43.51	9.88; 77.15	**<0.01**
Stability vs. Increase	20.67	−10.03; 51.38	0.24
**Pulmonary vascular volume**			
Decline vs. Stability	7.96	−11.11; 27.03	0.57
Decline vs. Increase	13.38	−11.59; 38.35	0.40
Stability vs. Increase	5.42	−17.38; 28.21	0.83
**Pulmonary vascular volume/Lung volume**			
Decline vs. Stability	10.98	−6.81; 28.77	0.30
Decline vs. Increase	27.54	4.25; 50.83	**0.02**
Stability vs. Increase	16.56	−4.7; 37.82	0.15
**Disease extent**			
Decline vs. Stability	10.22	−5.48; 25.92	0.27
Decline vs. Increase	26.33	5.78; 46.89	**<0.01**
Stability vs. Increase	16.12	−2.65; 34.88	0.11

## Data Availability

The original contributions presented in this study are included in the article/Supplementary Material. Further inquiries can be directed to the corresponding author.

## Supplementary Materials

The following supporting information can be downloaded at: https://www.mdpi.com/article/10.3390/diagnostics16101413/s1, Table S1. Baseline and longitudinal characteristics according to CTD subtype (SSC = Systemic Sclerosis; RA = Rheumatoid Artritis).

## Abbreviations

The following abbreviations are used in this manuscript:

CTD	Connective Tissue Disease
ILD	Interstitial Lung Disease
ILD-CTD	Interstitial Lung Disease Associated with Connective Tissue Disease
SSc	Systemic Sclerosis
RA	Rheumatoid Arthritis
SLE	Systemic Lupus Erythematosus
SS	Sjögren’s Syndrome
MCTD	Mixed Connective Tissue Disease
IPF	Idiopathic Pulmonary Fibrosis
OP	Organizing Pneumonia
BIP	Bronchiolocentric Interstitial Pneumonia
NSIP	Nonspecific Interstitial Pneumonia
HRCT	High-Resolution Computed Tomography
UIP	Usual Interstitial Pneumonia
FVC	Forced Vital Capacity
DLCO	Diffusing Capacity of the Lung for Carbon Monoxide
FU1/FU2	Follow-up 1 and 2
HU	Hounsfield Units
P90	Percentile 90 of lung voxel attenuation
R5	Percent of Lung Parenchyma with Attenuation Between −2000 and −700 HU
PACS	Picture Archiving and Communication System
YACTA	Yet Another CT Analyzer (software)
CII	Computerized Integrated Index
CALIPER	Computer-Aided Lung Informatics for Pathology Evaluation and Rating
ATS	American Thoracic Society
ERS	European Respiratory Society
JRS	Japanese Respiratory Society
ALAT	Latin American Thoracic Association
