# Supplementary material for: Quantitative Tomographic Analysis as a Prognostic Tool in Connective Tissue Disease-Associated Interstitial Lung Disease
Source: Diagnostics (Basel). 2026 May 7;16(10):1413. doi: 10.3390/diagnostics16101413 (PMC13205909; doi:10.3390/diagnostics16101413)
Supplement: Supplementary file 1 [file diagnostics-16-01413-s001.zip › diagnostics-4174844-supplementary.pdf]

diagnostics

MDPI

Supplementary Table S1. Baseline and longitudinal characteristics according to CTD subtype (SSC = Systemic Sclerosis; RA = Rheumatoid Artritis)

Variable	SSc (n=106)	RA (n=32)	Other CTDs (n=57)	p-value
**BASELINE**				
Age (years)	44.8 ± 11.9	58.7 ± 10.8	49.9 ± 12.6	<0.001
FVC (% predicted)	72.1 ± 17.5	74.3 ± 18.2	71.9 ± 16.8	0.74
DLCO (% predicted)	61.5 ± 22.1	63.2 ± 23.0	60.8 ± 21.7	0.82
Disease extent (%)	22.4 ± 9.1	24.1 ± 10.3 2	3.5 ± 9.8	0.56
Lung volume (cm^3)	4120 ± 980	4050 ± 1020	3890 ± 950	0.31
Mean lung density (HU)	-698 ± 45	-685 ± 48	-692 ± 46	0.22
**FOLLOW-UP 1**				
FVC (% predicted)	75.0 ± 19.8	77.3 ± 21.1	74.2 ± 18.7	0.68
DLCO (% predicted)	60.8 ± 22.5	62.1 ± 23.8	60.5 ± 21.9	0.91
Δ Lung volume (%)	-3.2 ± 6.5	-3.8 ± 7.1	-3.5 ± 6.8	0.79
Δ Disease extent (%)	+2.1 ± 5.4	+2.8 ± 5.9	+2.5 ± 5.7	0.71
Δ Mean lung density (HU)	+4.5 ± 10.2	+5.8 ± 11.4	+5.1 ± 10.8	0.66
**FOLLOW-UP 2**				
FVC (% predicted)	72.3 ± 20.5	73.8 ± 22.2	71.5 ± 19.9	0.84
DLCO (% predicted)	63.1 ± 21.4	64.2 ± 22.7	62.5 ± 21.0	0.88
Δ Lung volume (%)	-6.8 ± 8.9	-7.5 ± 9.6	-7.2 ± 9.1	0.77
Δ Disease extent (%)	+5.6 ± 7.8	+6.3 ± 8.4	+6.0 ± 8.1	0.73
Δ Fibrosis index (%)	+4.2 ± 6.5	+4.8 ± 7.1	+4.5 ± 6.8	0.81

At baseline, no significant differences were observed in quantitative CT parameters or pulmonary function tests between groups, except for age. Importantly, longitudinal analyses at both follow-up timepoints (approximately 12 and 60 months) demonstrated similar patterns of change across all subgroups, including comparable reductions in lung volume and increases in disease extent and fibrosis-related parameters.

No statistically significant differences were observed between CTD subtypes at any timepoint.

Authors acknowledge that some subgroups have limited sample sizes, which may reduce statistical power, representing a limitation of this analysis.

Event though, these findings support the robustness and generalizability of the results despite the heterogeneity of CTD subtypes.
